# Pathological Insights on Polypropylene Mesh Complications From Laparoscopic Sacrocolpopexy: A Case Series

**DOI:** 10.7759/cureus.56354

**Published:** 2024-03-17

**Authors:** Nobuo Okui, Machiko A Okui

**Affiliations:** 1 Dentistry, Kanagawa Dental University, Kanagawa, JPN; 2 Urology, Yokosuka Urogynecology and Urology Clinic, Kanagawa, JPN; 3 Urogynecology, Yokosuka Urogynecology and Urology Clinic, Kanagawa, JPN

**Keywords:** polypropylene mesh, mesh removal, laparoscopic sacrocolpopexy, pelvic organ prolapse, transvaginal natural orifice transluminal endoscopic surgery

## Abstract

Background

The use of polypropylene mesh in laparoscopic sacrocolpopexy (LSC) is a common treatment for pelvic organ prolapse (POP). Despite its widespread application, postoperative complications such as mesh pain and infection sometimes necessitate the removal of the mesh. However, it remains unclear in which cases mesh removal is warranted. Our research focused on the pathological changes at the sacral fixation point of the mesh. We sought to evaluate the pathological alterations of the sacral mesh removed through an innovative approach of transvaginal natural orifice transluminal endoscopic surgery (vNOTES).

Methods

This retrospective study included nine patients who underwent mesh removal surgery at the Yokosuka Urogynecology and Urology Clinic in 2023. Extraction surgery was performed using vNOTES with the GelPoint Access Platform (Applied Medical JAPAN HEADQUARTERS, Tokyo, Japan). Non-ablative Erbium YAG and Neodymium YAG lasers (RenovaLase, SP Dynamis; Fotona d.o.o., Ljubljana, Slovenia) were utilized for persistent stress urinary incontinence, fecal incontinence, vaginal erosion, and bleeding after surgery. Patients were categorized based on mesh fixation conditions, including unintended mesh overlap (Group I), excessive traction (Group II), and signs of mesh aging (Group III). This categorization helped to understand the distinct pathological outcomes associated with each condition.

Results

Pathological findings from the mesh removed via vNOTES varied significantly across the groups. In Group I, characteristic large vacuole formation and accumulation of atypical giant cells were observed, attributed to mesh overlap. Group II presented with vacuole formation, fiber degradation, and tissue destruction as a result of excessive mesh traction. In Group III, the aging of the mesh was marked by cracks in the surrounding tissues and granuloma formation. These detailed observations provide crucial insights into the underlying causes of mesh-related pain and other complications, highlighting the complexity of bodily responses to mesh implants.

Conclusion

This study demonstrated the effectiveness of vNOTES for polypropylene mesh removal in patients with post-LSC complications, resulting in significant pain reduction. Pathological analysis revealed that mesh-related issues stem from the surgical techniques, mesh properties, and long-term bodily reactions. These findings provide valuable insights for improving mesh design and POP treatment strategies. Despite the technical challenges, vNOTES is recommended for mesh removal in patients with pain. Additionally, the combination of UEL, VEL, targeted laser irradiation, AEL, and Nd:YAG laser treatments showed promising results in managing post-mesh removal complications such as stress urinary incontinence, vaginal erosion, bleeding, and fecal incontinence, offering hope for improved patient outcomes.

## Introduction

Pelvic organ prolapse is a condition in which the muscles and tissues of the pelvic floor weaken, causing the vagina and uterus to drop from their normal positions. This condition can significantly reduce women’s quality of life (QoL). In recent years, laparoscopic sacrocolpopexy (LSC) using polypropylene mesh has become widely popular as a treatment method for pelvic organ prolapse [1-10]. LSC is a minimally invasive surgical procedure and has become one of the mainstream methods for treating pelvic organ prolapse. In this surgical technique, it is common to place a mesh at the sacral promontory as part of the treatment for pelvic organ prolapse [2]. Adverse events associated with LSC include lower back pain [3,4], buttock pain [3,5], lumbar spondylitis and epidural abscess [6], postoperative urinary incontinence [7], and osteomyelitis [8]. These complications may affect the QoL of patients after surgery [10].

Investigating the causes of these symptoms is crucial, and pathology plays an essential role. However, research on the pathology of the mesh placed at the sacral colpopexy site in LSC is lacking in previous studies. Understanding the long-term effects and degradation of mesh, as well as the body's reaction to it, remains limited [11-13]. Further research in this area is necessary to improve treatment methods [14].

Vaginal Natural Orifice Transluminal Endoscopic Surgery (vNOTES) is a novel approach for addressing these challenges [15]. By employing vNOTES, surgeons gain direct access and visualization from the vagina to the sacral colpopexy site, which simplifies the mesh removal. This method enables effective extraction of problematic meshes with minimal invasiveness [15-19]. Utilizing this technique for mesh removal could result in shorter recovery periods and an enhanced quality of life for patients. Moreover, vNOTES not only benefits patients, but also allows for delicate dissection that provides sufficient tissue samples from the sacral fixation site for further examination.

In this study, we performed mesh removal via vNOTES in female patients with complications after undergoing LSC and conducted a detailed analysis of the pathology of the extracted mesh. While previous research on polypropylene mesh has identified evidence of adverse reactions associated with degradation of vaginal mesh fibers, our study focused on the mesh at the sacral colpopexy site, aiming to expand knowledge in this area.

## Materials and methods

Research design and ethical approval

This study was a retrospective analysis of patients who underwent polypropylene mesh removal surgery via vNOTES at the Yokosuka Urogynecology & Urology Clinic from January to December 2023. This study was approved by the ethics committee of our clinic. All patients provided written informed consent after being fully informed of the treatment before proceeding.

Participants

The study population was limited to women aged > 20 years who had previously undergone pelvic organ prolapse repair via LSC at other facilities and had experienced complications. Exclusion criteria included patients without a follow-up record for three months post-surgery, patients with severe immunodeficiency, and patients lacking the capacity to consent.

Surgical method

**Figure 1:**
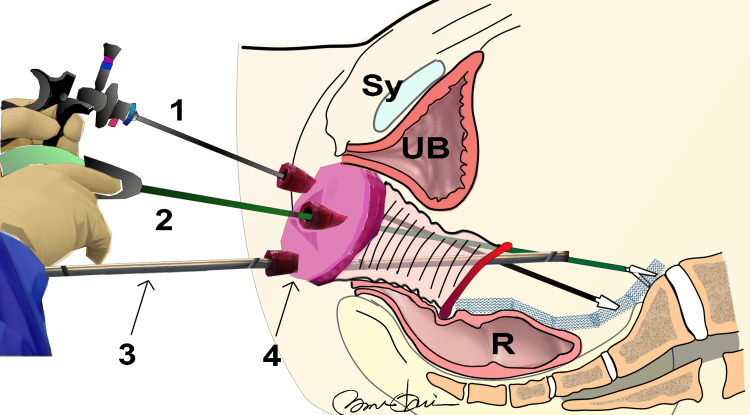
Mesh Removal via vNOTES UB: Bladder, Sy: Pubis, R: Rectum. 1: Ultrasonic coagulation cutting device SONICBEAT, 2: HICURA type grasping forceps, 3: Endoscope by OLYMPUS, 4: GelPoint™ Access Platform Illustration by Nobuo Okui

In this study, polypropylene mesh removal surgery was performed using vNOTES (Figure 1). This innovative technique involves transvaginal access of the natural orifice to remove the mesh [15]. The surgeon prepared the vNOTES for six months.

The surgery was performed under general anesthesia, with the patient in a supine, slight Trendelenburg position. At the beginning of the procedure, a 14 Fr urinary catheter was inserted, and 2 g cefazolin was administered intravenously for antibiotic prophylaxis. The surgeon positioned themselves in the patient's groin area, whereas the first assistant was positioned on the patient's right side. A clinical engineer was placed on the patient's left hand. A flexible monitor was placed in front of the surgeon [20].

In the first stage, a longitudinal incision was made in the center of the anterior and/or posterior vaginal wall, following the conventional vaginal mesh removal method of carefully separating the mesh from the surrounding tissue while preserving the important blood vessels and fascia. A hydrodissection device was used in cases with a risk of organ damage. Depending on the necessity, meshes from tension-free vaginal tape (TVT) or trans-obturator tape (TOT) [13,21] were carefully removed by thinning the vaginal wall.

In the second stage, for cases of hysterectomy, a 360-degree incision was made around the cul-de-sac where the uterus had previously been located, ensuring arterial blood flow. In cases where the uterus remained, incisions were made in the anterior and posterior vaginal fornices, completing a 360-degree loop. The GelPoint™ Access Platform (Applied Medical JAPAN HEADQUARTERS, Tokyo, Japan) [15,22], an OLYMPUS endoscopic ultrasonic coagulation cutting device SONICBEAT, and HICURA type grasping forceps (Olympus Medical Systems Corp., Tokyo, Japan) were introduced. The GelPoint™ device was inserted and, while maintaining a CO2 gas pressure of 8 mmHg [15], a standard 10 mm, 0° rigid laparoscope was inserted through the camera trocar at the 6 o'clock position, and work was performed using forceps and scissors inserted through the other two trocars.

In the third stage, the mesh was removed laparoscopically. When inspecting the incision of the peritoneal wall at the bladder-vaginal mesh connection, the delicacy of the tissue necessitates careful dissection. Strategic small incisions allowed for the separation and extraction of the mesh from the sacral surrounding tissues and posterior peritoneal wall, particularly in areas where the patient experienced discomfort. After mesh removal, the GelPoint device was removed through a vaginal incision. Furthermore, the mesh was carefully detached from the sacral fixation area to avoid damage to the nerves and vessels after preparing for a possible switch to open surgery. Especially in the sacral area, where vascular variants are common, dissection was performed under special light observation using the endoscopic videoscope system EVIS LUCERA SPECTRUM with Narrow Band Imaging system (Olympus Medical Systems Corp.), which emits light at wavelengths (540-560 nm) absorbed by hemoglobin in blood, highlighting vessels and mucosa in different hues [23].

In the final stage, two angled sutures were passed through the edges of the sacrospinous ligament identified in the second stage and the anterior and posterior vaginal walls were sutured. This step, known as Native Tissue Repair (NTR) [24], aimed to prevent the recurrence of pelvic organ prolapse and thoroughly restore and strengthen the patient's own tissue.

Measures for complications

**Figure 2:**
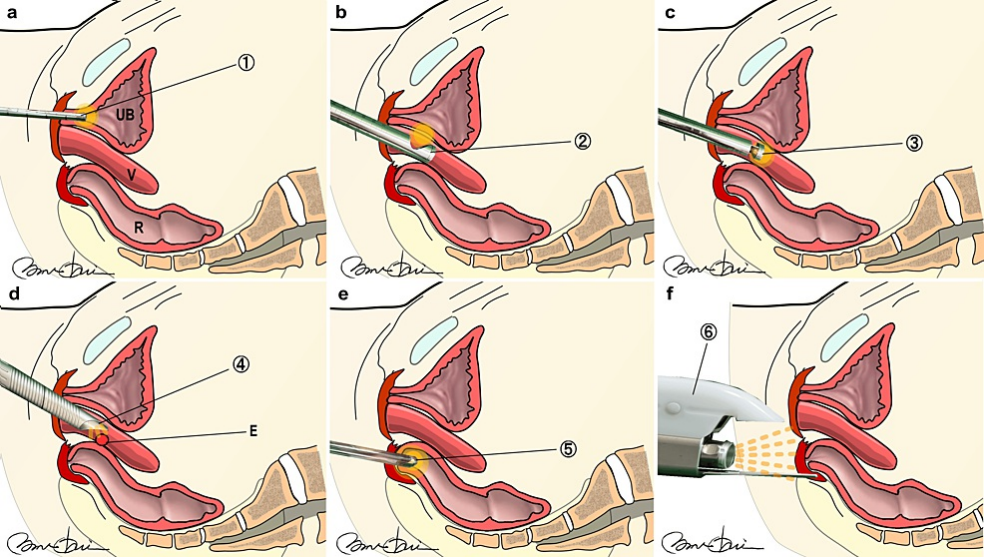
Non-ablative Erbium YAG and Neodymium YAG Laser Treatments a: UEL step (whole urethral laser irradiation with R09-2Gu) b: VEL step (laser irradiation of anterior vaginal wall by PS03) c: VEL step (whole vaginal laser irradiation by R11) d: Targeted irradiation of erosions step (indicated by red circle using the PS03) e: AEL step (circumferential intra-rectal Er:YAG irradiation with LA adapter) f: Nd:YAG laser (Fotona SP Dynamis, R33 non-contact handpiece, PIANO pulse mode). 1: R09-2Gu intraurethral Adapter, 2: PS03 90° angular golden mirror titanium adapter, 3: R11 360° circular golden mirror titanium adapter, 4: PS03 patterned titanium handpiece, 5: SmoothTouch LA Adapter, 6: R33 non-contact handpiece UB: Bladder, V: Vagina, R: Rectum, E: Erosions, UEL: Urethral Erbium:YAG laser, AEL: Anal Erbium:YAG laser, VEL: Vaginal Erbium YAG, NdYAG: Neodymium YAG Illustration by Nobuo Okui.

Complications considered after surgery included stress urinary incontinence, urge urinary incontinence, fecal incontinence, and vaginal bleeding, accompanied by erosion. Patients with stress urinary incontinence were presented with two options: adding TVT surgery during the vNOTES procedure or improving urinary incontinence without adding TVT through vaginal and urethral non-ablation erbium YAG laser (VEL + UEL) treatment [25]. If normalization of the vaginal mucosa was slow after surgery and erosion and bleeding persisted, VEL (Vaginal Erbium Laser) treatment was proposed [26]. Patients with fecal incontinence were offered two choices: receiving guidance on pelvic floor muscle exercises or improving fecal incontinence without additional surgery through vaginal and anal nonablative Erbium YAG laser and Neodymium YAG laser (VEL + AEL + Nd:YAG) treatment [27]. Figure 2 shows all the laser treatments.

For the UEL, after the withdrawal of residual urine from the bladder via a catheter, an R09-2 Gu laser probe designed for the urethra was used. The handpiece was connected to an Erbium YAG laser (SP Dynamis Fotona d.o.o, Ljubljana, Slovenia). The laser treatment settings were R09-2 Gu, SMOOTH, 1.4 Hz, 1.5 J/cm^2^, and four stacks from the urethral meatus to the proximal end in 2.5 mm increments (Figure 2a). This treatment was repeated four times.

For VEL, laser irradiation was performed using the same laser, starting with VEL and proceeding to UEL. The prepared devices included a special glass vaginal speculum dedicated to laser probes and handpieces (PS03 and R11, respectively). Each handpiece was connected to an SP dynamometer laser. In the VEL step, a glass speculum was inserted into the vagina, and the anterior vaginal wall was scanned with a PS03 laser probe with a spot size of 7 mm, pulse fluence of 6 J/cm^2^, and frequency of 2.0 Hz (Figure 2b). The area was irradiated at 5 mm intervals. This procedure was repeated thrice. Subsequently, the R11 laser probe was used to apply laser treatment at 5 mm intervals along the entire 360-degree vaginal canal. This treatment utilized a spot size of 7 mm, pulse fluence of 3.00 J/cm^2^, and frequency of 2.0 Hz (Figure 2c). This procedure was repeated twice.

For targeted laser irradiation, SMOOTH modality of Erbium laser was used to treat erosions on the posterior vaginal wall. Following the completion of the VEL procedure using handpieces connected to the SP Dynamis laser, a PS03 handpiece was employed to specifically target eroded areas. The settings for this treatment included a spot size of 7 mm, a pulse fluence of 6 J/cm^2^, and a frequency of 2.0 Hz. Although Figure 2d illustrates a schematic diagram with erosions indicated by red circles, the same protocol was applied, irrespective of the location of the erosions within the vagina.

For AEL, the anal sphincter width was measured via ultrasound before inserting the SmoothTouch LA Adapter attached to the R11 handpiece, applying 3.00 J/cm^2^, 2.0 Hz, 7 mm spot size in six segments once over the entire anal area for two minutes (Figure 2e).

For Nd:YAG laser treatment, a wavelength of 1064 nm was used in continuous PIANO pulse mode for 30 min. The non-contact R33 handpiece was set at an energy density of 90 J/cm^2^, with a five-second pulse duration and a spot size of 9 mm. The target areas for this treatment were the perineum and the external genitalia (Figure 2f).

Clinical evaluation

Pain was assessed using a Visual Analog Scale (VAS) [13,15]. For patients with urinary incontinence, evaluations were conducted using the 1-hour pad test [13,28], the International Consultation on Incontinence Questionnaire-Short Form (ICIQ-SF) [13], and the Overactive Bladder Symptom Score (OABSS) [13]. Patients with fecal incontinence were assessed using the St. Mark's Score [27].

Pathological evaluation method

**Figure 3:**
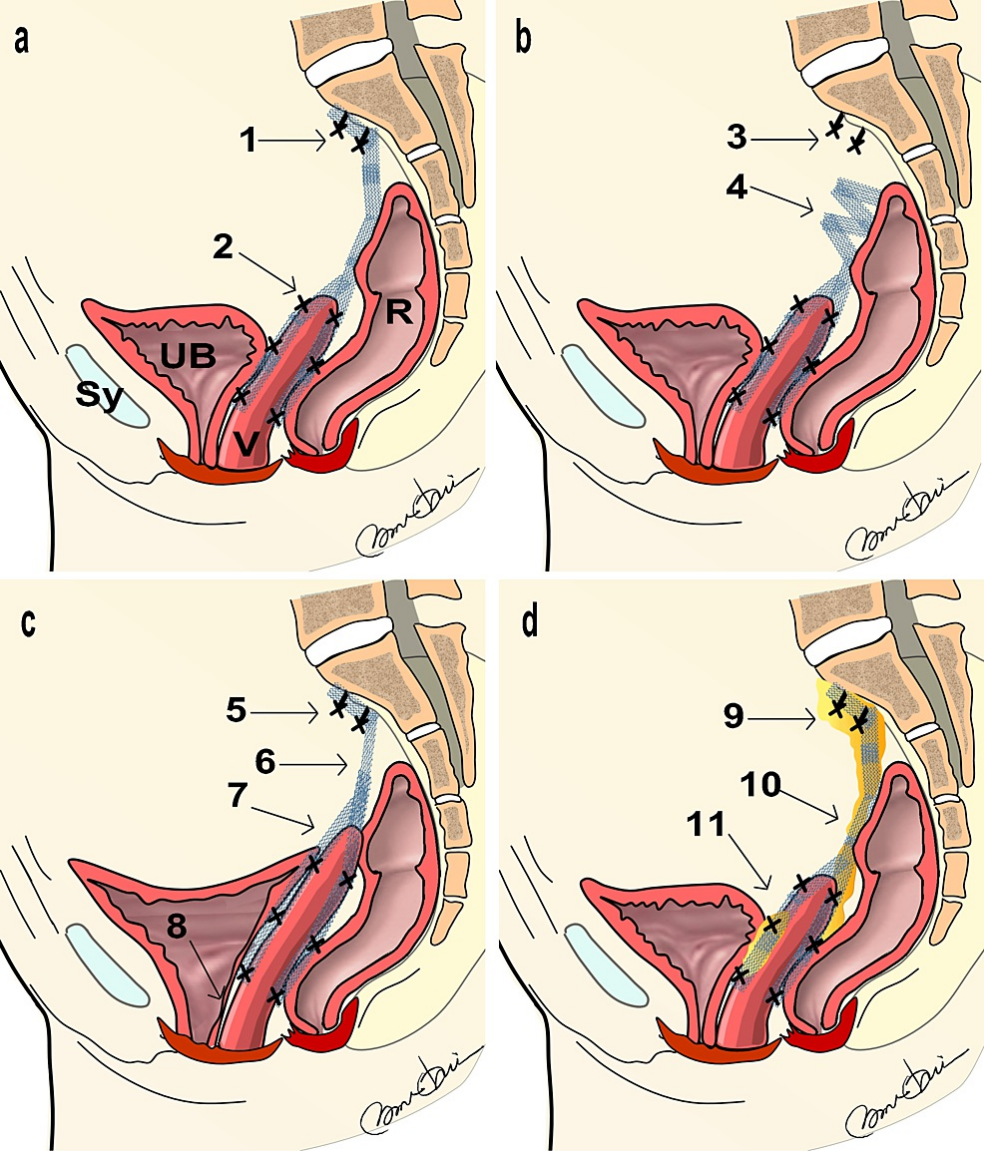
Three Groups of Mesh Complications in Polypropylene Mesh via LSC a: Illustration of polypropylene mesh inserted as planned. b: Illustration of polypropylene mesh where the sacral fixation has come off and formed adhesions in an unintended area (Group I). c: Illustration of polypropylene mesh that is either too short or has excessive traction (Group II). d: Illustration of polypropylene mesh that has aged and generated granulation tissue around it (Group III). 1: Sacral fixation area, 2: Vaginal stump fixation area, 3: Sacral fixation area (dislodged), 4: Area where polypropylene mesh overlaps and forms clumps, 5: Sacral fixation area (too high), 6: Area with strong traction on polypropylene mesh causing buckling, 7: Vaginal stump fixation area, 8: Condition where the urethra is pulled due to strong mesh traction, 9: Area with granulation tissue at the sacral fixation, 10: Area with granulation tissue at the border with the rectum, 11: Area with granulation tissue at the vaginal stump fixation. LSC: Laparoscopic Sacrocolpopexy Illustration by Nobuo Okui.

When extracting the mesh via vNOTES, the specimens were categorized into three groups. Figure 3a illustrates the insertion of the mesh via LSC. This illustration shows that the mesh was sutured to the anterior and posterior ends of the vaginal stump after hysterectomy, and the ends of the mesh were sutured to the tissue around the sacrum [29-31]. Figure 3b represents Group I, where the sacral fixation was detached and adhered to an unintended location. Pathological specimens were identified as cases in which the ends of the mesh overlapped and adhered to an unintended part. Figure 3c shows Group II, characterized by strong traction of the mesh from sacral fixation to the vaginal stump or to the bladder/rectum. Figure 3d represents Group III, where the LSC was fixed at the intended location but had undergone aging degradation. For Groups II and III, the study focused on the pathology of the sacral fixation area. Pathological samples were obtained from position 4 in Figure 3b, position 5 in Figure 3c, and position 9 in Figure 3d.

Statistical analysis

The data were analyzed using Python (Python Software Foundation, Wilmington, DE, USA) with the assistance of ChatGPT (OpenAI, San Francisco, CA, USA). Continuous variables were presented as mean ± standard deviation, and categorical variables were presented as frequencies and percentages. Comparisons between groups were made using the t-test or chi-square test, with a P-value <0.05 considered statistically significant.

## Results

Patient characteristics

From January to December 2023, 35 patients with POP who underwent polypropylene mesh insertion were observed at our clinic's specialized mesh complication service. Ten female patients had their meshes inserted via the LSC. Ten patients consented to mesh removal via vNOTES. In 2023, surgery was postponed in one patient because of myocardial infarction. Nine patients were treated using vNOTES.

Table 1 outlines the characteristics of the nine patients. The division into three groups was retrospectively based on the findings of vNOTES. The overall mean age was 75.67 ± 6.44 years, with Group I having the lowest mean age at 68.00 ± 1.41 years, Group II at 73.00 ± 4.58 years, and Group III being the oldest at 81.50 ± 2.08 years. Group II had the highest mean BMI at 29.50 ± 1.67 kg/m^2^, followed by Group I with a mean BMI of 26.60 ± 3.25 kg/m^2^, and Group III with a mean BMI of 25.17 ± 3.58 kg/m^2^.

**Table 1:** Characteristics of Patients Undergoing Mesh Removal via vNOTES LSC: Laparoscopic Sacrocolpopexy; SUI: Stress Urinary Incontinence; TVT: Tension-free Vaginal Tape; MUI: Mixed Urinary Incontinence; vNOTES: Vaginal Natural Orifice Transluminal Endoscopic Surgery; BMI: Body Mass Index

Patient Number	Age	BMI	Diagnosis at LSC	Features of LSC Surgery	Symptoms	Years since LSC (years)	vNOTES Surgery Time and Blood Loss	Group based on findings in vNOTES
1	67	24	Uterine Prolapse	Hysterectomy	Right back pain, Right buttock pain	1	90 mins, 55ml	I
2	69	26.2	Cuff Dehiscence	TVT combined	Right back pain	2	100 mins, 88ml	I
3	78	28	Uterine Prolapse & SUI	Hysterectomy. TVT added 3 months after LSC	Back pain, Urethral pain, MUI	2	90 mins, 58ml	II
4	72	29.2	Cuff Dehiscence	LSC only	Back pain	7	120 mins, 98ml	II
5	69	31.3	Uterine Prolapse	Hysterectomy & TVT combined	Back pain, MUI	8	150 mins, 130ml	II
6	82	27.9	Cystocele and Uterine Prolapse	Hysterectomy	Left back pain	6	80 mins, 80ml	III
7	79	28.4	Cuff Dehiscence	TVT combined	Back pain, Urethral pain	6	130 mins, 100ml	III
8	84	23.4	Cystocele and Uterine Prolapse	Hysterectomy & TVT combined	Back pain, Fecal incontinence	7	120 mins, 100ml	III
9	81	21	Cuff Dehiscence	Hysterectomy	Right back pain	8	120 mins, 65ml	III

Four patients underwent LSC surgery for vaginal cuff dehiscence following hysterectomy, and five patients underwent LSC at the same time as hysterectomy. Four patients had concurrent TVT and LSC as a prophylactic measure. One patient underwent TVT surgery after LSC surgery. The average duration from mesh insertion to treatment was 5.33 ± 2.60 years, with Group I having the shortest average duration at 2.00 ± 0 years, Group III the longest at 6.75 ± 0.96 years, and Group II at an average of 5.67 ± 3.21 years.

vNOTES and mesh removal

During the vNOTES procedure, Case 1 had a mesh twisted and fixed below the sacral fixation. In Case 2, the mesh was adhered 5 cm below the sacral fixation site. TVT was simultaneously removed in Cases 3 and 7. The two patients from whom the TVT was removed did not wish for TVT postoperatively due to stress urinary incontinence (SUI). Patient 7 opted for VEL + UEL postoperatively. The reason for these choices was their concern over the mesh issues they experienced this time.

The average surgical time was 111.11 ± 22.61 minutes overall, with Group I having the shortest average time at 95.00 ± 7.07 minutes, Group II at 120.00 ± 30.00 minutes, and Group III at 112.50 ± 22.17 minutes. The average intraoperative blood loss was 86.00 ml ± 24.23 ml overall. Group I had the least average blood loss at 71.50 ml ± 23.33 ml, Group III at 86.25 ml ± 17.02 ml, and Group II had the most at 95.33 ml ± 36.07 ml.

Pathological examination

**Figure 4:**
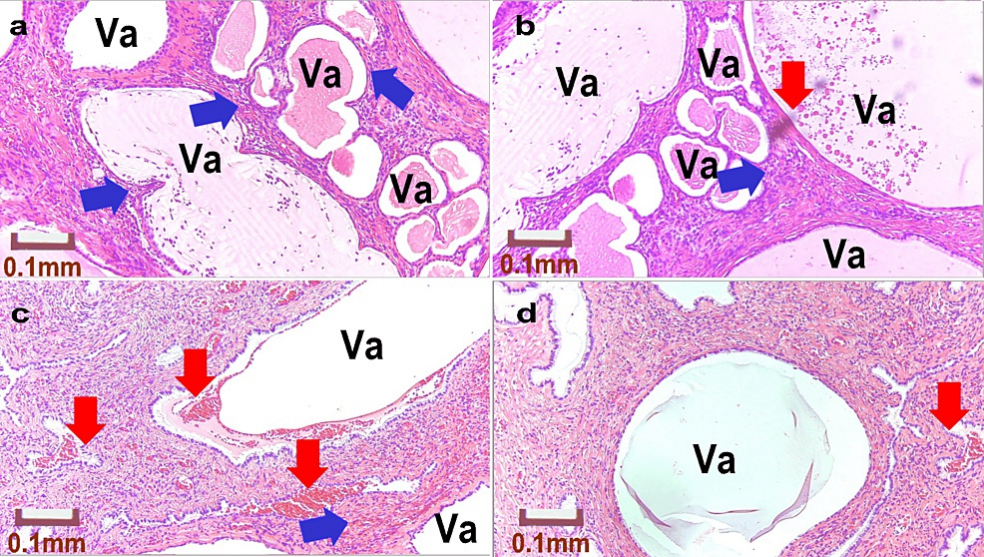
Pathology of the Sacral Suture Site in Group I a: Case 1; b: Case 1; c: Case 2; d: Case 2 Va: Vacuoles formed by polypropylene mesh fibers, Blue arrows: Aggregates of foreign body giant cells, Red arrows: Hemorrhage All images were stained with Hematoxylin and Eosin (H&E) and captured at an optical magnification of 40x using the Olympus System Microscope BX43 and objective lens LPLN10X (Olympus Corporation, Tokyo, Japan).

Figure 4 shows the pathological specimens of the two patients in Group I. In Case 1 (Figure 4a, 4b), overlapping polypropylene fibers were observed, forming large vacuoles (Va). In contrast, Case 2 (Figure 4c, 4d) displayed bleeding around the large vacuoles.

**Figure 5:**
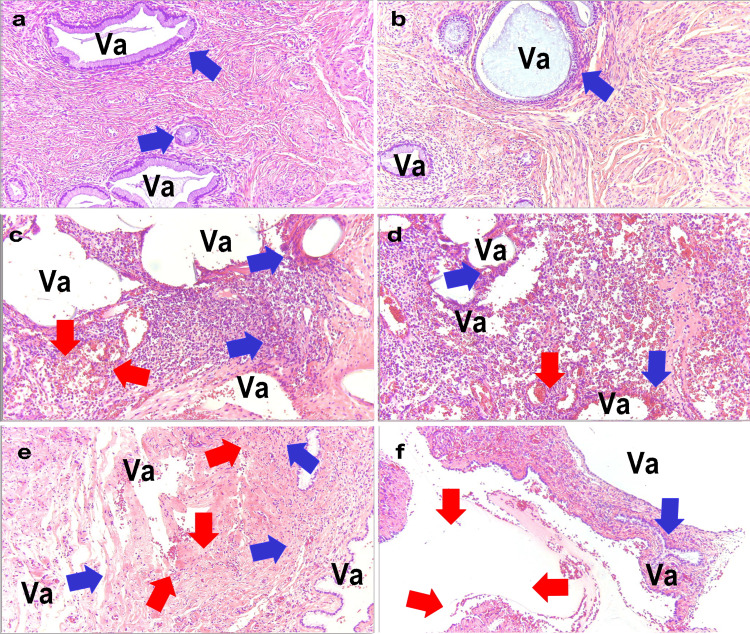
Pathology of the Sacral Suture Site in Group II a: Case 3; b: Case 3; c: Case 4; d: Case 4; e: Case 5; f: Case 5 Va: Vacuoles formed by polypropylene mesh fibers, Blue arrows: Aggregates of foreign body giant cells, Red arrows: Hemorrhage All images were stained with Hematoxylin and Eosin (H&E) and captured at an optical magnification of 40x using the Olympus System Microscope BX43 and objective lens LPLN10X (Olympus Corporation, Tokyo, Japan).

Figure 5 shows the pathological specimens of the three patients in Group II. In Case 3 (Figure 5a, 5b), despite the strong traction on the polypropylene mesh, the vacuoles, which are traces of fibers, were uniform. In addition, as it was only one year post-surgery, the tissue was densely present around the fibers. A small number of foreign-body giant cells are observed (blue arrows). However, in Case 4 (Figure 5c, 5d), seven years had passed, and the tissue around the fibers was damaged owing to the degradation of the fibers, which were fraying and destroying the surrounding tissue. Additionally, some vacuoles merged. Numerous hemorrhages (red arrows) and abscesses (blue arrows) were observed. Case 5 (Figure 5e, 5f) showed advanced aging degradation of the fibers, with numerous cracks in the tissue surrounding the fibers. Therefore, vacuoles (Va) were unable to maintain their shape. In particular, in areas with the highest tension, most of the tissue was lost. The remaining scant tissue shows signs of hemorrhage (red arrows) and abscess formation (blue arrows).

**Figure 6:**
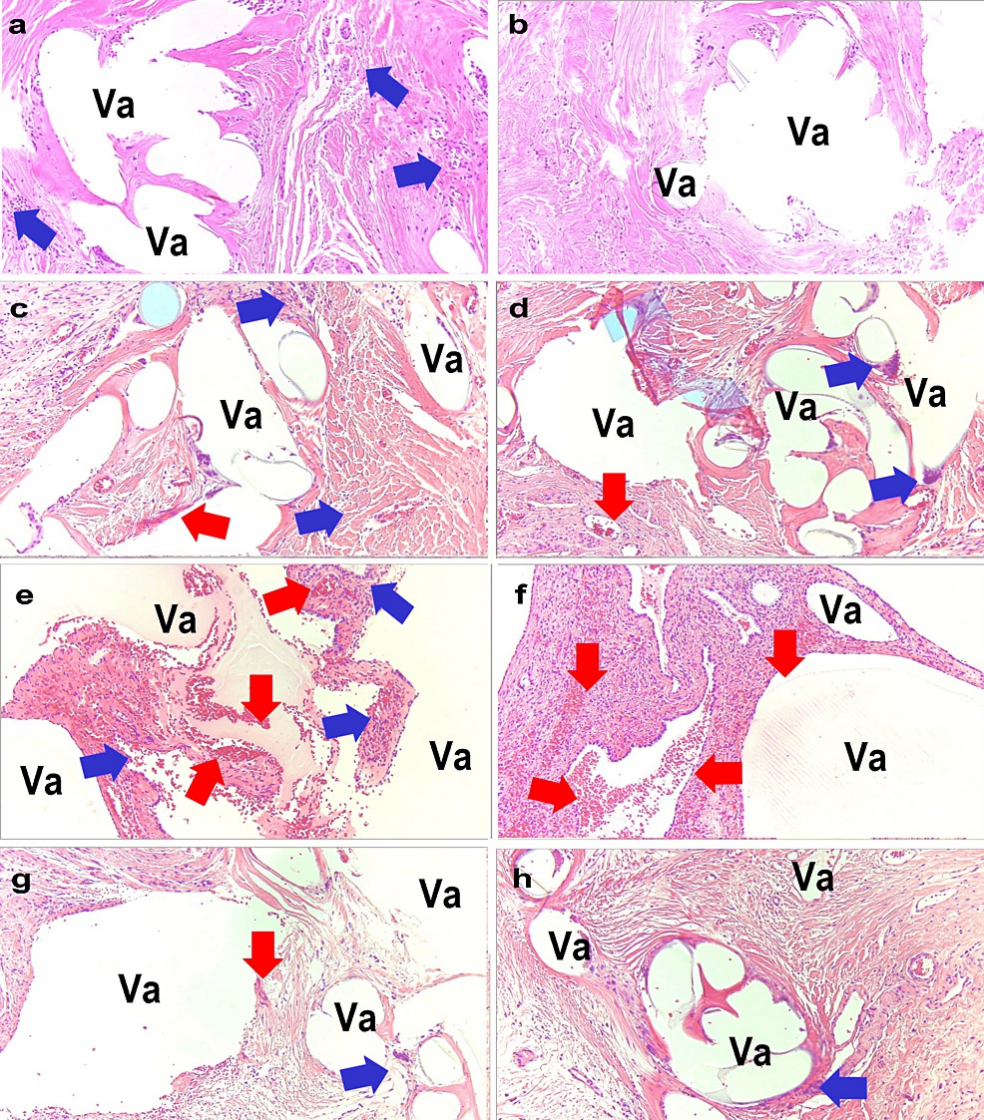
Pathology of the Sacral Suture Site in Group III a: Case 6; b: Case 6; c: Case 7; d: Case 7; e: Case 8; f: Case 8; g: Case 9; h: Case 9 Va: Vacuoles formed by polypropylene mesh fibers, Blue arrows: Aggregates of foreign body giant cells, Red arrows: Hemorrhage All images were stained with Hematoxylin and Eosin (H&E) and captured at an optical magnification of 40x using the Olympus System Microscope BX43 and objective lens LPLN10X (Olympus Corporation, Tokyo, Japan).

Figure 6 presents the pathological specimens of the four patients in Group III. Case 6 (Figure 6a, 6b) showed degradation of the polypropylene mesh fibers owing to long-term placement, resulting in fraying. This caused vacuoles to become destroyed and enlarged. There were areas almost devoid of tissue around the fibers. Foreign body giant cells were observed (blue arrows). In Case 7 (Figure 6c, 6d), the fibers were degraded, decimating the surrounding tissue. Foreign body giant cells formed granulation tissue (blue arrows) and hemorrhages (red arrows). Case 8 (Figure 6e, 6f) demonstrated vacuoles that had collapsed, and the tissue had almost entirely disappeared. Case 9 (Figure 6g, 6h) showed vacuoles that had merged and enlarged owing to the aging degradation of the fibers (Va), with some tissue loss. Hemorrhage (red arrows) and abscess formation (blue arrows) were evident.

Clinical effects of mesh removal

The study evaluated the preoperative and three-month postoperative pain levels. Across all nine cases, the average VAS score decreased from 9.0 ± 0.71 preoperatively to 1.22 ± 0.97 postoperatively. For Group I (Cases 1 and 2), the average preoperative VAS was 9.5 ± 0.71, decreasing to 0.5 ± 0.71 postoperatively. In Group II (Cases 3, 4, and 5), the mean preoperative VAS average was 8.33 ± 0.58, reducing to 0.33 ± 0.58 postoperatively. Group III (Cases 6, 7, 8, 9) saw a decrease in the average VAS from 9.25 ± 0.5 preoperatively to 1.25 ± 1.26 postoperatively.

Case 1's buttock and back pain improved simultaneously with the removal of overlapped and dislodged polypropylene mesh, which was thought to be caused by tension preventing pelvic organ prolapse. The TVT mesh was removed because of Mixed Urinary Incontinence. The ICIQ-SF score improved from 9 preoperatively to 2 postoperatively. The OABSS score improved from 9 preoperatively to 2 postoperatively.

Case 3 showed improvement in SUI, with the ICIQ-SF score changing from 4 preoperatively to 0 postoperatively and the OABSS score from 4 to 0. Case 3 did not experience recurrence of SUI, suggesting that the cause of SUI post-LSC was the strong traction from the polypropylene mesh.

Case 6 had bleeding from vaginal erosion prior to treatment. If the patient's initial assessment was 10, after the mesh removal, the vaginal erosion did not completely disappear but was reduced, with some bleeding remaining, leading the patient to rate it a 4. After conducting three sets of VEL treatments (once a month) for one month, the bleeding completely disappeared, and the patient's rating went down to 0.

Case 7, whose urethral pain improved after mesh removal, developed SUI, which then improved with VEL + UEL treatment. The ICIQ-SF score changed from 4 before mesh removal to 8 after mesh removal and 1 after laser treatment.

Cases 8 and 9 required significant time for the removal of the polypropylene mesh adhered to the bladder. Case 8 did not show symptoms of overactive bladder (OAB) preoperatively or postoperatively. The St. Mark's score for mucous fecal incontinence improved from 4.0 preoperatively to 2.0 postoperatively. After a single treatment with VEL + AEL + Nd:YAG, Case 8 experienced complete disappearance of fecal incontinence, with the St. Mark's score reaching 0.

**Figure 7:**
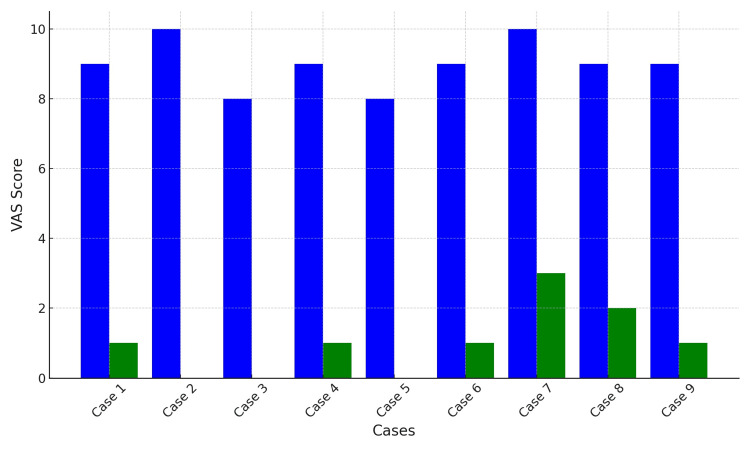
Comparison of Pain Scores Before and Three Months After Surgery Using VAS The vertical axis represents the VAS scores, which range from 0 to 10. The horizontal axis lists each patient (Cases 1–9). The graph displays the VAS scores before surgery (blue bars) and three months after surgery (green bars) for each patient. The VAS is used to quantify the intensity of pain on a scale from 0 (no pain) to 10 (worst possible pain). VAS: Visual analog scale.

Case 9 developed mild symptoms of OAB postoperatively, with the OABSS score increasing from 1 preoperatively to 4 postoperatively, indicating a transition to mild OAB. The symptoms were successfully resolved with the administration of 50 mg of Vibegron (Figure 7).

## Discussion

First, the discussion focuses on the pain associated with mesh surgery. Complications associated with LSC include back pain [1,2], buttock pain [1], lumbar spondylitis and epidural abscess [3], and osteomyelitis [4]. Chuang et al. reported that after LSC, 19% of patients experienced back pain and 4.8% experienced buttock pain [3]. Claerhout et al. reported mesh erosion at 4.5% and mesh-related pain at 2.3% following LSC [5]. Sato et al. reported a 2.2% incidence of retroperitoneal abscess and 2.2% worsening of stress urinary incontinence after LSC surgery [7]. There appears to be variability in the reported incidence of complications [32].

This study targeted patients who experienced pain after LSC, primarily those who reported lower back and buttock pain. No cases of lumbar inflammation, epidural abscess, or osteomyelitis were noted. Based on previous research and this study, it is speculated that lower back and buttock pain are common side effects. Regardless of the frequency of occurrence, it is believed that these pains are treatable and, therefore, should be targeted for treatment.

Second, the current status of mesh extraction is examined. Mohr et al. emphasize the significance of lower back pain resulting from mesh erosion in LSC and indicate the necessity for new techniques in managing complications [6]. Apostolis and Heiselman reported a case of osteomyelitis that developed after tooth extraction in a patient who underwent LSC [8], highlighting the challenges of systemic management in patients who underwent LSC surgery. However, the incidence of reoperation after LSC is surprisingly low, with Vandendriessche et al. reporting rates of surgery related to urinary incontinence of 5.5%, surgery for POP recurrence of 5.1%, and mesh-related surgery of 2.8% [10,33].

This study, in conjunction with the presented evidence, shows that mesh removal surgery carries potential risks. This necessitates an approach that is minimally invasive towards the eroded mesh part and minimizes the risk of infection compared to traditional open surgery. Should such techniques be developed, it is anticipated that mesh-related reoperations will become more widespread.

Third, mesh complications are analyzed from a pathological perspective. The most important aspect of surgical treatment is the mechanism by which the polypropylene mesh causes pain. Pathological studies on polypropylene mesh by Klosterhalfen et al. showed that long-term implantation leads to a persistent inflammatory foreign body reaction in the host tissue, which could lead to chronic wounds or malignant transformations [11]. Additionally, research by Dievernich et al. indicated that chronic foreign body reaction to polypropylene mesh is associated with M2 macrophage response, collagen deposition, and MMP-2 expression, challenging the traditional belief that M1 macrophages are primarily responsible for inflammation [12]. Furthermore, prior research on pain following mid-urethral sling (MUS) procedures for urinary incontinence has identified unhealthy granulation tissue around the mesh [13].

This study revealed distinct characteristics for each of the three groups when comparing the pathology based on the mechanism by which the polypropylene mesh causes pain. In Group I, the sacral fixation became detached, leading to the mesh being fixed in an unintended overlap, preventing cellular regeneration in the overlapped spaces of the mesh in the pathology. In Group II, excessive tension at the sacral fixation site hindered tissue regeneration. The findings in Groups I and II highlight issues with the surgical technique itself related to mesh pathology. In Group III, it is believed that the aging of the polypropylene mesh fibers caused cracks in the surrounding tissues and promoted the development of unhealthy granulation tissue, indicating issues related to the material properties of the mesh revealed by pathology.

Fourth, the vNOTES surgical technique for mesh extraction is evaluated. vNOTES has been reported to have numerous advantages over traditional open surgery, including minimal invasiveness, reduced postoperative pain, and faster recovery [34-37]. Additionally, it has been shown to be beneficial in patients with a high body mass index [16,17]. Furthermore, it is anticipated that vNOTES, which allows for the direct visualization of problematic mesh areas through the vagina, will be a more effective approach than laparoscopic mesh removal because of the latter's limited visibility of the affected areas. vNOTES also offers a minimally invasive approach compared to traditional open surgery, reducing the risk of infection and postoperative complications [34-37]. Moreover, vNOTES, which allows direct visualization and manipulation of the mesh through the vaginal canal, may offer better access to the mesh than abdominal laparoscopic approaches. These advantages of vNOTES are expected to improve patients' QoL [34].

This study considered vNOTES to be a promising surgical method for several reasons [15-19]. First, in our clinical experience, there is currently no consensus regarding the most effective method for mesh removal. Second, vNOTES did not result in postoperative fever or pelvic infections. This study also found that the mean surgical time for polypropylene mesh removal using vNOTES was 111.11 ± 22.61 minutes. Although there are currently no directly comparable studies on the surgical time for polypropylene mesh removal, these results suggest that vNOTES is an efficient surgical method for mesh removal. Overall, our findings highlight the potential of vNOTES as a safe and minimally invasive alternative to address complications arising from mesh implantation in LSC.

One of the challenges of vNOTES is the high level of skill required for proficiency [16]. Furthermore, mesh removal carries risks of ureteral and rectal injuries [15]. Therefore, to expand the use of vNOTES for mesh removal, it is essential to address these challenges through technical advancements [19].

This study required a six-month training period before initiating mesh removal using vNOTES. Additionally, prior experience with numerous mesh removal procedures proved valuable [25].

Fifth, the consideration of reinsertion after mesh extraction is addressed. The French clinical practice guidelines emphasize the importance of carefully evaluating the necessity of mesh reinsertion for POP reconstruction after mesh removal. This is because specific subgroups of patients are at a higher risk of complications following POP surgery with synthetic mesh [38]. According to these guidelines, surgical mesh removal should be considered if complications occur after POP surgery. However, the decision to use mesh in subsequent POP surgeries after mesh removal should be made cautiously, considering the patient's specific circumstances and the risks associated with mesh use [38].

This study primarily employed NTR techniques, considering the patients' history of complications and decision to undergo mesh removal [24]. NTR techniques, such as sacrospinous ligament fixation and uterosacral ligament suspension, can provide adequate support to the pelvic organs and prevent POP recurrence without mesh reinsertion [24]. The use of NTR techniques in this study aligns with the French guidelines' recommendation of exercising caution when considering mesh use in POP surgeries after mesh removal. However, it is important to note that there is limited evidence regarding the optimal POP treatment approach following mesh removal. Although NTR techniques have shown promise in preventing POP recurrence, the long-term effectiveness of these methods compared to mesh reinsertion remains unclear. As stated in the French guidelines, it is crucial to develop a comprehensive plan for POP treatment after mesh removal, considering the individual patient's circumstances and the potential risks associated with mesh use.

Finally, the resolution of complications following mesh extraction is explored. Recent studies have shown significant advancements in the treatment of complications after mesh removal, including laser therapy. The current study demonstrated the effectiveness of UEL and VEL for stress urinary incontinence following mesh removal [13]. This finding is supported by previous studies that have shown tissue normalization following VEL + UEL treatment, suggesting that the combination of MUS removal and VEL + UEL treatment may be an effective option for managing stress urinary incontinence. Furthermore, UEL + VEL may contribute to the improvement of pelvic floor muscle function, providing an additional benefit for patients [25,39].

VEL and targeted laser irradiation have been histopathologically proven to effectively manage vaginal erosion and associated bleeding in previous study [26]. The results of the present study corroborate these findings, highlighting the potential of VEL and targeted laser irradiation as novel treatment options for vaginal erosion and bleeding after mesh removal. These laser treatments offer a promising solution for patients who struggle with these complications after mesh removal.

Previous studies have established the efficacy of AEL, VEL, and Nd:YAG laser treatments for vaginal atrophy (VVA) and anal incontinence (AI) in postmenopausal women [27,40]. These laser treatments improve insufficient blood flow in pelvic vessels through a process known as "re-canalization." Notably, Nd:YAG lasers have been shown to enhance blood flow in the perineum and external genitalia. The combination of AEL, VEL, and Nd:YAG laser treatments may provide a promising therapeutic approach for fecal incontinence following mesh removal, offering hope for patients seeking alternative treatment options.

This study, leveraging our past experiences [13,25-27], contributed to symptom alleviation in all patients without immediate mesh insertion.

This study's limitations include a small sample size and brief follow-up, leading to limited statistical power, warranting caution in generalizing findings. The success of vNOTES, a novel surgical technique, may largely depend on the surgeon's expertise. The findings enhance understanding of long-term reactions and impacts from polypropylene mesh use, underscoring the need for more research on its effects, degradation, and the body's response, to improve patient care and outcomes.

## Conclusions

This study presents polypropylene mesh removal using vNOTES in patients who experienced pain following treatment with POP and LSC. vNOTES significantly reduced pain in patients with complications related to the polypropylene mesh. Pathological analysis of tissues meticulously dissected via vNOTES revealed that issues associated with the mesh vary due to surgical techniques, physical properties of the mesh, and long-term reactions within the body. These insights offer valuable perspectives for future mesh design improvements and the selection of POP treatment methods.

Although mesh removal surgery requires consideration of technical limitations and the skill set required for mastery, pathological findings indicate that removal should be pursued in patients with pain, recommending vNOTES as an effective method. Furthermore, the combination of laser treatments, including UEL, VEL, targeted laser irradiation, AEL, and Nd:YAG, has shown promising results in managing complications, such as stress urinary incontinence, vaginal erosion, bleeding, and fecal incontinence following mesh removal.
